# Co-application of ACC-deaminase producing PGPR and timber-waste biochar improves pigments formation, growth and yield of wheat under drought stress

**DOI:** 10.1038/s41598-019-42374-9

**Published:** 2019-04-12

**Authors:** Subhan Danish, Muhammad Zafar-ul-Hye

**Affiliations:** Department of Soil Science, Faculty of Agricultural Sciences and Technology, Bahauddin Zakariya University, Multan, 60800 Punjab Pakistan

## Abstract

Besides other deleterious effects, drought elevates ethylene level too in plants. Increased ethylene concentration reduces root elongation and development that consequently retard plant growth and yield. There are certain PGPR which produce ACC-deaminase. The ACC-deaminase converts ACC (an immediate precursor of ethylene biosynthesis in methionine pathway in higher plants) into ammonia and α-ketobutyrate instead of ethylene. Regularization of ethylene level in plants mitigate the effects of drought. On the other hand, biochar has been reported to be rich in nutrients and exhibiting higher water holding capacity. So, a pot study was conducted with the hypothesis that the combined application of ACC-deaminase producing PGPR and biochar would minimize the drought effects on wheat growth. The ACC-deaminase producing PGPR were applied on wheat seeds in combination with two biochar doses. Three moisture levels were maintained throughout the trial. The data obtained revealed that *B*. *amyloliquefaciens* + 2BC improved the chlorophyll a, chlorophyll b, photosynthetic rate, transpiration rate, 100-grain weight, and grain N, P and K up to 114%, 123%, 118%, 73%, 59%, 58%, 18% and 23%, respectively, under drought conditions. It is concluded that co-application of PGPR and biochar is an effective technique to mitigate the drought effects.

## Introduction

Various biotic (pests, pathogens) and abiotic (soil compaction, drought, salinity, waterlogging, heavy metals, poor nutrition etc.) stresses are a big cause of low crops productivity around the globe^1^. Drought stress is very common in worldwide arid and semi-arid areas. Moreover, climate change is going to create the worst situation in this regard^2–6^. The demand for irrigation water is expected to increase by 10% up to 2050^7^. Under drought stress, growth and yield of crops are usually decreased due to less intake of nutrients, poor photosynthesis^8^ and limited supply of water^9^. In addition, drought accelerates the biosynthesis of ethylene^10,11^ which retards the roots elongation and development^12–14^.

Although, traditional breeding, water management and genetic engineering are thought to be useful tools to alleviate drought stress but high technicalities are involved to adopt and implement these approaches^15^. However, the use of plant growth promoting rhizobacteria (PGPR) is an alternative technique for mitigation of drought effects^15^. A large number of rhizospheric bacteria are well documented that show growth promotion in plants under stressful conditions^14^. As far as regularization of ethylene biosynthesis under drought stress is concerned, using 1-aminocyclopropane-1-carboxylate deaminase (ACC-deaminase) producing PGPR is found to be quite effective^16–20^. The ACC-deaminase cleaves the ACC (1-aminocyclopropane-1-carboxylic acid, an immediate precursor of ethylene biosynthesis through methionine pathway in higher plants) into ammonia and α-ketobutyrate instead of ethylene^11,21,22^. Besides ethylene regularization, the PGPR also help in better root development^23^, secretion of growth hormones (auxins or cytokinins)^22^ and solubilization of immobile nutrients (phosphorus, potassium etc.)^24,25^.

On the other hand, the imperative role of organic amendments in mitigation of drought stress by improving soil water holding capacity and availability of nutrients cannot be denied^26,27^. Biochar (BC), is a black carbon compound which is a good source of nutrients. It is produced through pyrolysis at high temperature under low or no supply of oxygen^27–29^. The physio-chemical properties of BC depend on the nature of waste material used and temperature of the pyrolysis^30,31^. High surface area and pore spaces of BC-structure improve soil water and nutrients holding capacity^32–35^.

Wheat (*Triticum aestivum* L.) is an important cereal crop and staple food in most parts of the world. It contains 55% carbohydrates and 8–12% proteins^36^. It is an important crop due to its worldwide trade too^37^. Cultivation of wheat under a limited supply of water significantly decreases the yield^38^ while its demand is increasing at the rate of 1.6%/annum^39^. The need of time is to enhance wheat yield even in the areas under drought stress.

In recent past, the researchers focused on the application of either ACC-deaminase containing PGPR or BC in separate to mitigate the drought stress. The novelty and aim of the present study are to examine the combined effect of ACC-deaminase producing PGPR and timber-waste BC for the alleviation of drought effects. Keeping in mind the importance of wheat, the current study was conducted with the hypothesis that the co-application of drought tolerant ACC deaminase producing PGPR and timber waste BC could be very effective to alleviate drought effects.

## Results

### Gas exchange attributes

Main effects of treatments (T) and various levels of drought (D) were significantly different, while their interaction (T × D) remained similar for the rate of photosynthesis and transpiration. For stomatal conductance, both main and interactive effects of T and D were significantly different. The photosynthetic rate was significantly improved as compared to the control where *P*. *aeruginosa* and *B*. *amyloliquefaciens* were applied (Table 1). The BC application without PGPR significantly enhanced photosynthetic rate as compared to the control but the application rate 2BC was more effective than 1BC for the improvement in photosynthetic rate. However, among all the treatments *P*. *aeruginosa* + 2BC and *B*. *amyloliquefaciens* + 2BC remained the best to significantly increase the photosynthetic rate. For transpiration rate, a significant improvement was noted as compared to the control in all the treatments. Application of 1BC and 2BC without PGPR gave statistically similar results regarding transpiration rate. However, *P*. *aeruginosa* + 2BC and *B*. *amyloliquefaciens* + 2BC remained significantly better for transpiration rate as compared to *L*. *adecarboxylata*, *A*. *fabrum*, *P*. *aeruginosa*, *B*. *amyloliquefaciens*, 1BC and the control. Maximum increase in photosynthetic rate (118%) and transpiration (73%) rate was noted as compared to control where *B*. *amyloliquefaciens* + 2BC was applied. Application of 1BC and 2BC remained significantly better at severe drought (SD) level as compared to the control for stomatal conductance. However, at mild drought (MD) level, application of 2BC remained significantly better than 1BC and control regarding stomatal conductance. The *B*. *amyloliquefaciens* + 2BC at SD level while 2BC, *P*. *aeruginosa* + 2BC and *B*. *amyloliquefaciens* + 2BC at MD level performed significantly better among other treatments for stomatal conductance. However, stomatal conductance was maximum as compared to the control through 2BC (56%), *B*. *amyloliquefaciens* + 2BC (146%) and *B*. *amyloliquefaciens* + 2BC (5.62-fold) at normal moisture (NM), MD and SD levels_,_ respectively.

**Table 1:** Effect of ACC deaminase containing PGPR in combination with various rates of timber waste biochar (0BC, 1BC and 2BC) on gas exchange attributes under various levels of drought (D).

Treatments	Photosynthetic Rate [µmol (CO_2_) m^−2^ s^−1^]				Transpiration Rate (mmol (H_2_O) m^−2^ s^−1^)				Stomatal Conductance mol (CO_2_) m^−2^ s^−1^			
	Various levels of drought (D)											
	IE (T × D) (Means of 3 replicates)			ME (T)	IE (T × D) (Means of 3 replicates)			ME (T)	IE (T × D) (Means of 3 replicates)			ME (T)
	NM	MD	SD		NM	MD	SD		NM	MD	SD	
Control_(No PGPR+No BC)_	35.9	22.0	16.0	24.6^G^	1.84	1.12	0.70	1.22^D^	35.7^e–l^	21.7^m–p^	6.70^r^	21.4^H^
*L*. *adecarboxylata*	44.1	32.6	17.7	31.5^FG^	1.98	1.40	0.89	1.42^CD^	39.8^d–j^	24.5^l–p^	8.71^qr^	24.3^GH^
*A*. *fabrum*	43.4	31.4	14.1	29.6^FG^	2.07	1.79	1.02	1.63^BC^	41.4^b–h^	28.9^h–n^	8.38^qr^	26.2^GH^
*P*. *aeruginosa*	46.7	38.0	21.2	35.3^D–F^	2.12	1.76	1.03	1.64^BC^	36.4^e–l^	26.0^k–n^	12.4^p–r^	24.9^GH^
*B*. *amyloliquefaciens*	46.9	38.2	27.4	37.5^C–F^	2.11	1.80	1.08	1.66^BC^	37.5^d–k^	29.0^h–n^	13.1°^–r^	26.5^F–H^
1BC	46.7	34.6	19.1	33.5^EF^	2.24	1.87	0.84	1.65^BC^	37.9^d–k^	32.2^e–n^	28.1^i–n^	32.7^EF^
*L*. *adecarboxylata* + 1BC	52.3	46.5	33.9	44.2^BC^	2.36	1.84	1.33	1.84^AB^	36.2^e–l^	28.5^i–n^	25.8^k–o^	30.1^E–G^
*A*. *fabrum* + 1BC	50.6	43.7	35.4	43.2^B–D^	2.43	1.80	1.31	1.85^AB^	37.4^d–l^	27.6^j–n^	20.1^n–q^	28.4^FG^
*P*. *aeruginosa* + 1BC	49.0	41.8	36.8	42.5^B–D^	2.41	1.79	1.32	1.84^AB^	39.2^d–j^	35.8^e–l^	32.2^e–n^	35.7^DE^
*B*. *amyloliquefaciens* + 1BC	48.3	43.3	33.6	41.7^B–E^	2.42	1.91	1.36	1.90^AB^	41.6^b–h^	35.2^e–l^	29.5^g–n^	35.4^DE^
2BC	52.5	45.2	37.9	45.2^BC^	2.40	1.89	1.39	1.89^AB^	55.8^a^	44.9^a–e^	39.9^d–j^	46.9^A–C^
*L*. *adecarboxylata* + 2BC	47.6	43.8	36.2	42.5^B–D^	2.40	2.03	1.32	1.92^AB^	55.7^a^	40.5^c–i^	33.8^e–m^	43.4^BC^
*A*. *fabrum* + 2BC	50.5	49.9	34.1	44.8^BC^	2.34	2.08	1.24	1.88^AB^	53.9^ab^	37.2^d–l^	31.8^f–n^	41.0^CD^
*P*. *aeruginosa* + 2BC	54.3	49.5	39.3	47.7^AB^	2.40	2.19	1.54	2.04^A^	55.0^a^	49.6^a–d^	41.9^b–g^	48.8^AB^
*B*. *amyloliquefaciens* + 2BC	62.9	58.6	39.4	53.6^A^	2.32	2.27	1.73	2.11^A^	55.3^a^	53.3^a–c^	44.2^a–f^	50.9^A^
°ME (D)	48.8^A^	41.3^B^	29.5^C^		2.26^A^	1.84^B^	1.21^C^		43.9^A^	34.3^B^	25.1^C^	

Means sharing different letters are significantly different (*P* ≤ 0.05). Non-significant interactive effect (T × D) did not have any letter.

ME indicates main effect; IE indicates interactive effect; NM = Normal Moisture; MD = Mild Drought; SD = Severe Drought.

### Shoot length and electrolyte leakage

**Figure 1:**
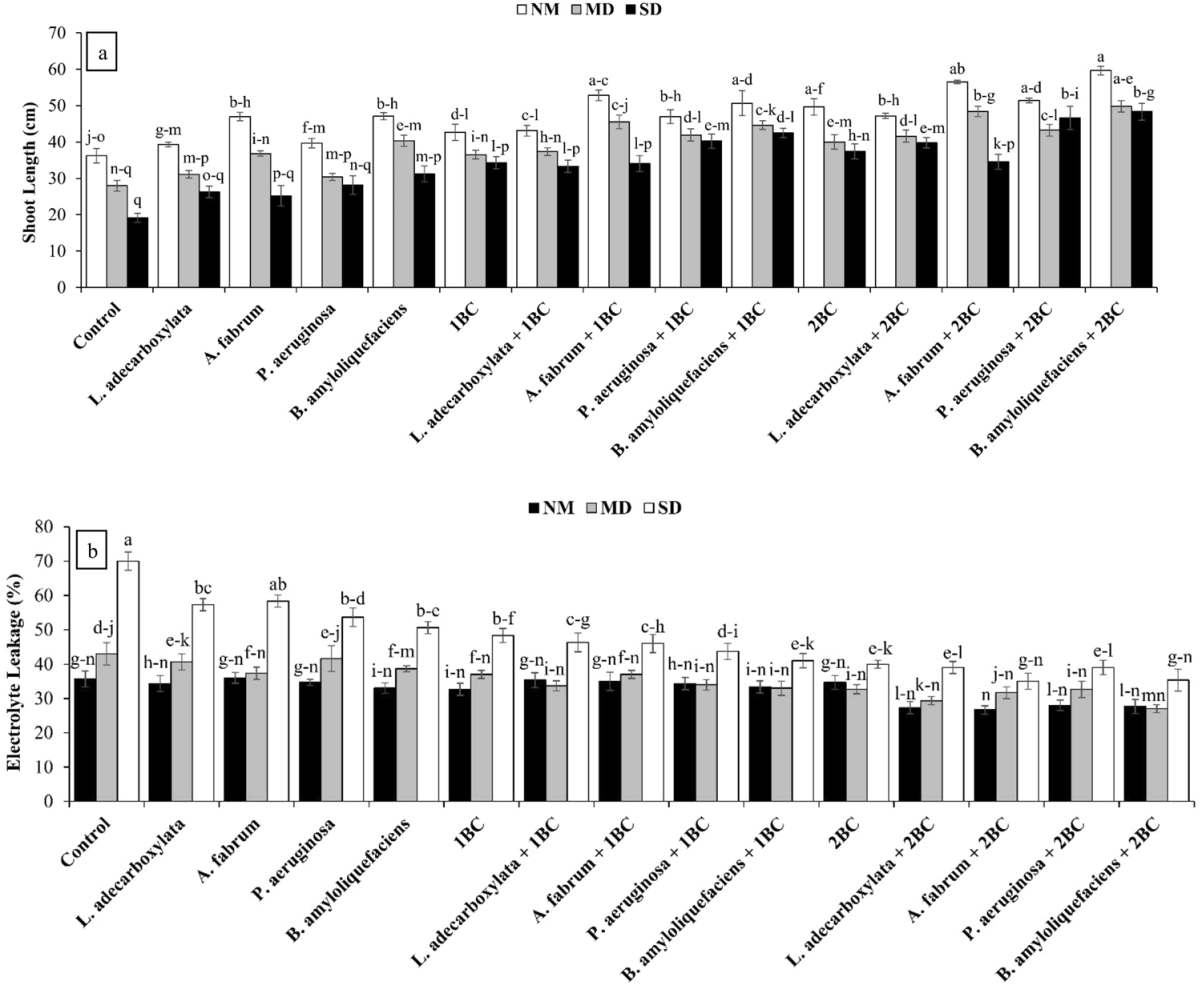
Effect of drought tolerant ACC deaminase containing PGPR and various levels of timber waste biochar on shoot length (**a**) and electrolyte leakage (**b**) in wheat leaves under various levels of drought (D). Means sharing the same letter are statistically similar. Error bars represent ± standard error. NM = Normal Moisture; MD = Mild Drought; SD = Severe Drought.

Both main and interactive effects of T and D were significantly different for shoot length and electrolyte leakage in wheat leaves. At SD level, the *B*. *amyloliquefaciens* + 2BC and *P*. *aeruginosa* + 2BC remained significantly better as compared to rest of the treatments for shoot length (Fig. 1). The 1BC and 2BC remained statistically alike but significantly different as compared to the control at SD for shoot length. However, *B*. *amyloliquefaciens* + 2BC also remained significantly better among other treatments for shoot length at MD level. Maximum increase, 153% and 73% in shoot length was noted at SD and MD levels respectively, as compared to the control where *B*. *amyloliquefaciens* + 2BC was applied (Fig. 1a). In case of electrolyte leakage at SD, *A*. *fabrum* + 2BC and *B*. *amyloliquefaciens* + 2BC were found to be significantly better as compared to the control. The 1BC and 2BC remained statistically alike but significantly different as compared to the control at SD for electrolyte leakage (Fig. 1b). The *L*. *adecarboxylata*, *P*. *aeruginosa*, *B*. *amyloliquefaciens* without BC also decreased the electrolyte leakage as compared to the control at SD. Maximum reduction (50%) in electrolyte leakage was noted as compared to the control where *A*. *fabrum* + 2BC and *B*. *amyloliquefaciens* + 2BC were applied at SD.

### Carotenoids and proline

**Figure 2:**
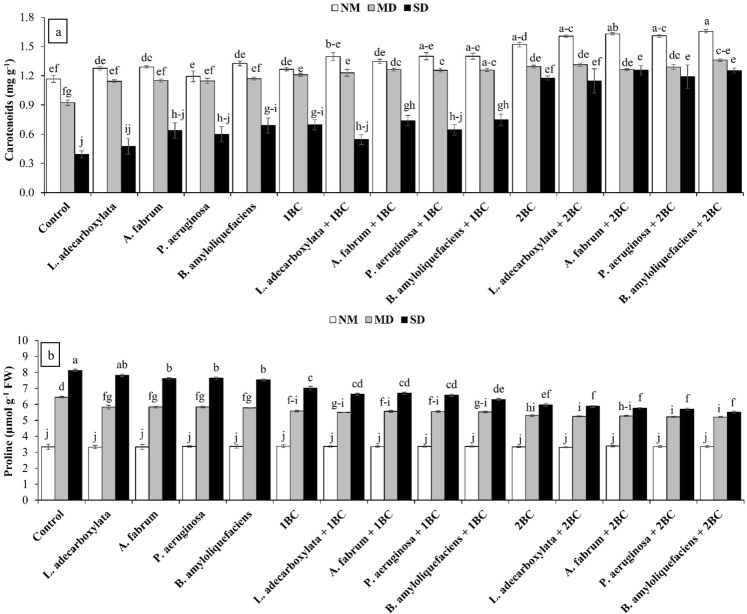
Effect of drought tolerant ACC deaminase containing PGPR and various levels of timber waste biochar on carotenoids (**a**) proline (**b**) in wheat leaves under various levels of drought (D). Means sharing the same letter are statistically similar. Error bars represent ± standard deviations. NM = Normal Moisture; MD = Mild Drought; SD = Severe Drought.

Both main and interactive effects of T and D were significantly different for carotenoids and proline contents. In case of carotenoids, the treatments, *L*. *adecarboxylata* + 2BC, *A*. *fabrum* + 2BC, *P*. *aeruginosa* + 2BC and *B*. *amyloliquefaciens* + 2BC were statistically alike but differed significantly as compared to the control for carotenoids at SD (Fig. 2). The 2BC proved significantly better as compared to 1BC and the control for carotenoids at SD. The 1BC, *L*. *adecarboxylata* + 1BC, *A*. *fabrum* + 1BC, *P*. *aeruginosa* + 1BC, *B*. *amyloliquefaciens* + 1BC, 2BC, *L*. *adecarboxylata* + 2BC, *A*. *fabrum* + 2BC, *P*. *aeruginosa* + 2BC and *B*. *amyloliquefaciens* + 2BC were statistically alike but significantly different as compared to the control for carotenoids at MD (Fig. 2a). Maximum increase i.e., 42%, 48% and 220% in carotenoids was noted at NM, MD and SD respectively as compared to the control where *B*. *amyloliquefaciens* + 2BC was applied. For proline reduction, *A*. *fabrum*, *P*. *aeruginosa* and *B*. *amyloliquefaciens* differed significantly as compared to the control at SD. The 1BC significantly decreased proline too as compared to control, but the 2BC was statistically far better for proline reduction as compared to 1BC at SD. The PGPR strains remained significantly better with 2BC for proline reduction as compared to with 1BC at SD (Fig. 2b). However, the *B*. *amyloliquefaciens* + 2BC showed maximum decrease in proline i.e. 34% at SD as compared to the control.

### Photosynthetic pigments

Main effects of T and D were significantly different but their interactions remained similar for chlorophyll a, chlorophyll b and total chlorophyll contents in wheat leaves. The treatments *L*. *adecarboxylata*, *A*. *fabrum*, *P*. *aeruginosa* and *B*. *amyloliquefaciens* were statistically alike but differed significantly as compared to the control for chlorophyll a content. With and without PGPR, the application of 1BC and 2BC gave statistically similar results but remained significantly different as compared to the control for chlorophyll a (Table 2). For chlorophyll b contents, with and without 1BC, all the PGPR treatments (except *B*. *amyloliquefaciens* + 1BC) were statistically alike but differed significantly as compared to the control. Without PGPR, the 2BC was significantly better than 1BC as compared to the control for chlorophyll b contents. However, *A*. *fabrum* + 2BC and *B*. *amyloliquefaciens* + 2BC proved significantly better as compared to other treatments regarding chlorophyll b contents. In case of total chlorophyll contents, the treatments *L*. *adecarboxylata*, *A*. *fabrum*, *P*. *aeruginosa* and *B*. *amyloliquefaciens* differed significantly as compared to the control. Statistically, 2BC was significantly better than 1BC for total chlorophyll. However, the 1BC and 2BC significantly increased total chlorophyll as compared to the control. The 2BC, *L*. *adecarboxylata* + 2BC, *A*. *fabrum* + 2BC, *P*. *aeruginosa* + 2BC and *B*. *amyloliquefaciens* + 2BC were statistically alike and proved better among the other treatments for total chlorophyll contents. Maximum increase in chlorophyll a (114%), chlorophyll b (123%) and total chlorophyll (115%) was noted through *B*. *amyloliquefaciens* + 2BC as compared to the control.

**Table 2:** Effect of ACC deaminase containing PGPR in combination with various rates of timber waste biochar (0BC, 1BC and 2BC) on photosynthetic pigments under various levels of drought (D).

Treatments	Chlorophyll a (mg g^−1^)				Chlorophyll b (mg g^−1^)				Total Chlorophyll (mg g^−1^)			
	Various levels of drought (D)											
	IE (T × D) (Means of 3 replicates)			ME (T)	IE (T × D) (Means of 3 replicates)			ME (T)	IE (T × D) (Means of 3 replicates)			ME (T)
	NM	MD	SD		NM	MD	SD		NM	MD	SD	
Control_(No PGPR+No BC)_	1.04	0.67	0.39	0.70^E^	0.29	0.26	0.10	0.22^G^	1.33	0.93	0.50	0.92^H^
*L*. *adecarboxylata*	1.21	0.92	0.78	0.97^D^	0.43	0.34	0.15	0.31^EF^	1.64	1.26	0.93	1.28^G^
*A*. *fabrum*	1.14	0.93	0.83	0.96^D^	0.40	0.38	0.12	0.30^EF^	1.54	1.31	0.95	1.27^G^
*P*. *aeruginosa*	1.09	0.92	0.78	0.93^D^	0.36	0.31	0.14	0.27^F^	1.45	1.24	0.93	1.20^G^
*B*. *amyloliquefaciens*	1.12	1.04	1.04	1.07^CD^	0.42	0.39	0.17	0.32^EF^	1.54	1.42	1.21	1.39^FG^
1BC	1.43	1.31	1.07	1.27^BC^	0.40	0.40	0.17	0.32^EF^	1.82	1.71	1.24	1.59^EF^
*L*. *adecarboxylata* + 1BC	1.58	1.36	1.17	1.37^AB^	0.43	0.38	0.18	0.33^E^	2.02	1.74	1.36	1.70^C–E^
*A*. *fabrum* + 1BC	1.42	1.38	1.29	1.36^AB^	0.42	0.40	0.22	0.35^DE^	1.84	1.78	1.52	1.71^C–E^
*P*. *aeruginosa* + 1BC	1.58	1.26	1.15	1.33^AB^	0.45	0.38	0.18	0.34^E^	2.03	1.64	1.33	1.67^DE^
*B*. *amyloliquefaciens* + 1BC	1.53	1.35	1.24	1.37^AB^	0.48	0.41	0.29	0.40^CD^	2.01	1.76	1.54	1.77^B–E^
2BC	1.60	1.42	1.17	1.40^AB^	0.51	0.47	0.26	0.41^BC^	2.10	1.89	1.44	1.81^A–D^
*L*. *adecarboxylata* + 2BC	1.64	1.37	1.30	1.44^AB^	0.51	0.44	0.28	0.41^BC^	2.14	1.81	1.58	1.85^A–D^
*A*. *fabrum* + 2BC	1.59	1.44	1.35	1.46^AB^	0.55	0.52	0.31	0.46^AB^	2.15	1.96	1.66	1.92^AB^
*P*. *aeruginosa* + 2BC	1.61	1.51	1.29	1.47 ^AB^	0.52	0.49	0.29	0.43^BC^	2.12	2.00	1.58	1.90^A–C^
*B*. *amyloliquefaciens* + 2BC	1.65	1.49	1.35	1.50^A^	0.56	0.51	0.38	0.49^A^	2.21	2.00	1.74	1.98^A^
ME (D)	1.41^A^	1.22^B^	1.08^C^		0.45^A^	0.41^B^	0.22^C^		1.86^A^	1.63^B^	1.30^C^	

Means sharing different letters are significantly different (*p* ≤ 0.05). Non-significant interactive effect (T × D) did not have any letter.

ME indicates main effect; IE indicates interactive effect; NM = Normal Moisture; MD = Mild Drought; SD = Severe Drought.

### N, P and K in shoot

Main effects of T and D were significantly different but their interaction remained similar for nitrogen (N) and phosphorus (P) concentration in shoot. For potassium (K) concentration in shoot, both main and interactive effects of T and D were significantly different. The treatments, *L*. *adecarboxylata*, *A*. *fabrum*, *P*. *aeruginosa* and *B*. *amyloliquefaciens* differed significantly as compared to the control for shoot N concentration. The 2BC remained significantly better as compared to 1BC for N concentration in shoot (Table 3). The PGPR (*L*. *adecarboxylata*, *A*. *fabrum*, *P*. *aeruginosa* and *B*. *amyloliquefaciens*) with 2BC performed significantly better as compared to 1BC (except *B*. *amyloliquefaciens* + 1BC) for N concentration in shoot. For phosphorus concentration in shoot, 1BC and 2BC significantly differed as compared to the control. In case of P concentration in shoot, *L*. *adecarboxylata*, *A*. *fabrum* and *B*. *amyloliquefaciens* were statistically alike but significantly different as compared to the control. The 2BC significantly increased (i.e., by 28%) P concentration in shoot as compared to 1BC. The treatments *A*. *fabrum* + 2BC, *P*. *aeruginosa* + 2BC and *B*. *amyloliquefaciens* + 2BC proved significantly better for P concentration in shoot. Maximum increase in N (73%) and P (150%) concentration in shoot was noted as compared to the control where *B*. *amyloliquefaciens* + 2BC was applied. In case of K concentration in shoot, all the treatments remained significantly better as compared to control at SD. The treatments 1BC and 2BC remained statistically alike without PGPR for K concentration in shoot at MD and SD. The *B*. *amyloliquefaciens* + 1BC, *A*. *fabrum* + 2BC, *P*. *aeruginosa* + 2BC and *B*. *amyloliquefaciens* + 2BC were significantly better for K concentration in shoot at MD. Maximum increase of 38%, 85% and 138% in K concentration in shoot was noted where *B*. *amyloliquefaciens* + 2BC was applied as compared to the control at NM, MD and SD, respectively.

**Table 3:** Effect of ACC deaminase containing PGPR in combination with various rates of timber waste biochar (0BC, 1BC and 2BC) on shoot N, P and K concentration under various levels of drought (D).

Treatments	Shoot Nitrogen (%)				Shoot Phosphorus (%)				Shoot Potassium (%)			
	Various levels of drought (D)											
	IE (T × D) (Means of 3 replicates)			ME (T)	IE (T × D) (Means of 3 replicates)			ME (T)	IE (T × D) (Means of 3 replicates)			ME (T)
	NM	MD	SD		NM	MD	SD		NM	MD	SD	
Control_(No PGPR+No BC)_	1.66	1.43	1.28	1.45^F^	0.29	0.20	0.12	0.20^H^	2.06^f–o^	1.44 ^r^	0.99^s^	1.50^I^
*L*. *adecarboxylata*	2.01	1.93	1.52	1.82 ^E^	0.37	0.24	0.21	0.27^FG^	2.11^e–o^	1.94^i–q^	1.62^p–r^	1.89^GH^
*A*. *fabrum*	2.08	1.95	1.64	1.89^DE^	0.37	0.25	0.22	0.28^FG^	2.13^e–o^	2.01^g–p^	1.57^qr^	1.90^F–H^
*P*. *aeruginosa*	2.06	1.93	1.56	1.85^E^	0.33	0.21	0.19	0.24^GH^	2.09^e–o^	1.88^k–q^	1.52^qr^	1.83^H^
*B*. *amyloliquefaciens*	2.05	1.97	1.63	1.88^DE^	0.39	0.26	0.18	0.27^FG^	2.15^e–n^	2.02^g–p^	1.71^o–r^	1.96^F–H^
1BC	2.24	2.13	1.85	2.07^CD^	0.45	0.30	0.22	0.32^EF^	2.29^c–l^	1.93^i–q^	1.73^n–r^	1.99^E–H^
*L*. *adecarboxylata* + 1BC	2.37	2.23	2.00	2.20^BC^	0.49	0.33	0.25	0.35^C–E^	2.37^b–i^	2.14^e–o^	1.77^n–r^	2.10^D–G^
*A*. *fabrum* + 1BC	2.47	2.26	1.94	2.22^BC^	0.52	0.36	0.30	0.39^B–D^	2.42^a–g^	2.32^c–k^	1.82^n–r^	2.19^C–E^
*P*. *aeruginosa* + 1BC	2.42	2.26	1.97	2.22^BC^	0.46	0.35	0.20	0.34^D–F^	2.34^c–j^	2.27^d–m^	1.72^n–r^	2.11^D–F^
*B*. *amyloliquefaciens* + 1BC	2.52	2.35	2.06	2.31^AB^	0.54	0.41	0.31	0.42^B^	2.50^a–f^	2.43^a–g^	1.84^m–r^	2.25^B–D^
2BC	2.59	2.48	1.90	2.32^AB^	0.55	0.39	0.28	0.41^BC^	2.45^a–g^	2.36^b–i^	1.92^j–q^	2.24^B–D^
*L*. *adecarboxylata* + 2BC	2.67	2.51	2.15	2.44^A^	0.53	0.42	0.31	0.42^BC^	2.68^a–d^	2.38^b–h^	1.86^l–r^	2.30^B–D^
*A*. *fabrum* + 2BC	2.72	2.55	2.06	2.44^A^	0.60	0.48	0.39	0.49^A^	2.79^ab^	2.61^a–d^	1.94^i–q^	2.44^AB^
*P*. *aeruginosa* + 2BC	2.77	2.57	2.13	2.49^A^	0.55	0.42	0.37	0.45^AB^	2.71^a–c^	2.53^a–e^	1.94^h–q^	2.39^BC^
*B*. *amyloliquefaciens* + 2BC	2.79	2.57	2.17	2.51^A^	0.61	0.49	0.40	0.50^A^	2.85^a^	2.67^a–d^	2.35^b–j^	2.63^A^
ME (D)	2.36^A^	2.21^B^	1.86^C^		0.47^A^	0.34^B^	0.26^C^		2.40^A^	2.20^B^	1.75^C^	

Means sharing different letters are significantly different (*p* ≤ 0.05). Non-significant interactive effect (T × D) did not have any letter.

ME indicates main effect; IE indicates interactive effect; NM = Normal Moisture; MD = Mild Drought; SD = Severe Drought.

### Yield attributes

Both main and interactive effects of T and D were significantly different for grain yield pot^−1^ and straw yield pot^−1^. In case of 100-grain weight, main effects of T and D were significantly different but their interaction remained statistically similar. The PGPR with and without 1BC, as well as, 1BC and 2BC were statistically at par with the control at SD for grain yield pot^−1^. However, the *L*. *adecarboxylata* + 2BC, *A*. *fabrum* + 2BC, *P*. *aeruginosa* + 2BC and *B*. *amyloliquefaciens* + 2BC differed significantly as compared to the control for grain yield pot^−1^ at MD and SD (Table 4). The treatments *B*. *amyloliquefaciens* + 1BC also remained significantly better as compared to the control and other PGPR with 1BC at MD for grain yield pot^−1^. At NM, the *B*. *amyloliquefaciens* + 1BC, 2BC, *L*. *adecarboxylata* + 2BC, remained significantly different as compared to the control for grain yield pot^−1^. Maximum increase, 40%, 155% and 215% in grain yield pot^−1^ was noted at NM, MD and SD as compared to the control where *L*. *adecarboxylata* + 2BC, *P*. *aeruginosa* + 2BC and *B*. *amyloliquefaciens* + 2BC were applied, respectively. For 100-grain weight, all the treatments (except *A*. *fabrum*) remained significantly better as compared to the control. The 1BC and 2BC remained statistically alike for 100-grain weight. The 1BC, *A*. *fabrum* + 1BC, *B*. *amyloliquefaciens* + 1BC, 2BC, *L*. *adecarboxylata* + 2BC, *A*. *fabrum* + 2BC, *P*. *aeruginosa* + 2BC and *B*. *amyloliquefaciens* + 2BC remained significantly better for 100-grain weight. Maximum increase (59%) in 100-grain weight was noted as compared to the control where *B*. *amyloliquefaciens* + 2BC was applied_._ In case of straw yield pot^−1^, the PGPR with and without BC remained significantly better as compared to the control at NM, MD and SD. However, 1BC and 2BC remained statistically alike for straw yield pot^−1^. The *L*. *adecarboxylata* + 1BC, *A*. *fabrum* + 1BC, *P*. *aeruginosa* + 1BC, *B*. *amyloliquefaciens* + 1BC, 2BC, *L*. *adecarboxylata* + 2BC, *A*. *fabrum* + 2BC, *P*. *aeruginosa* + 2BC and *B*. *amyloliquefaciens* + 2BC remained significantly better for straw yield pot^−1^. Maximum increase i.e., 181% and 178% in straw yield pot^−1^ was noted as compared to the control where *B*. *amyloliquefaciens* + 2BC and *A*. *fabrum* + 2BC were applied at SD and MD respectively.

**Table 4:** Effect of ACC deaminase containing PGPR in combination with various rates of timber waste biochar (0BC, 1BC and 2BC) on grains yield pot^−1^, 100-grains weight and straw yield under various levels of drought (D).

Treatments	Grain Yield Pot^−1^ (g)				100-grains weight (g)				Straw Yield Pot^−1^ (g)			
	Various levels of drought (D)											
	IE (T × D) (Means of 3 replicates)			ME (T)	IE (T × D) (Means of 3 replicates)			ME (T)	IE (T × D) (Means of 3 replicates)			ME (T)
	NM	MD	SD		NM	MD	SD		NM	MD	SD	
Control_(No PGPR+No BC)_	5.82^d–m^	2.93^q–u^	1.84^u^	3.53^E^	2.89	2.01	1.30	2.07^E^	15.3^h–n^	6.80^st^	4.70^t^	8.90^F^
*L*. *adecarboxylata*	6.03^b–k^	4.56^j–r^	2.73^r–u^	4.44^C–E^	2.89	2.80	2.26	2.65^CD^	16.1^g–l^	12.9^l–q^	9.50^q–s^	12.9^E^
*A*. *fabrum*	5.53^f–o^	3.34^p–u^	2.45^s–u^	3.77^DE^	3.04	2.47	1.95	2.49^DE^	16.7^f–k^	14.9^h–n^	10.6^p–r^	14.1^DE^
*P*. *aeruginosa*	5.74^e–n^	3.74^m–u^	2.17^tu^	3.88^DE^	3.00	2.83	1.90	2.58^CD^	16.1^g–l^	12.2^m–r^	9.20^rs^	12.5^E^
*B*. *amyloliquefaciens*	5.90^c–l^	3.87^l–u^	2.95^q–u^	4.24^DE^	3.12	2.81	2.19	2.71^B–D^	17.4^e–j^	14.8^i–n^	10.1^p–s^	14.1^DE^
1BC	6.69^a–i^	4.44^j–s^	2.87^q–u^	4.67^CD^	3.13	2.97	2.52	2.87^A–D^	18.5^e–h^	14.4^j–o^	9.10^rs^	14.0^DE^
*L*. *adecarboxylata* + 1BC	6.45^a–j^	4.76^i–r^	2.73^r–u^	4.65^CD^	3.07	2.88	2.43	2.79^B–D^	20.0^a–f^	16.7^f–k^	10.4^p–r^	15.7^CD^
*A*. *fabrum* + 1BC	7.21^a–g^	5.37^g–p^	3.70^n–u^	5.43^BC^	3.29	3.10	2.54	2.98^A–D^	21.1^a–d^	15.6^g–m^	12.0^n–r^	16.2^BC^
*P*. *aeruginosa* + 1BC	6.28^a–j^	4.90^h–q^	2.87^q–u^	4.68^CD^	3.11	2.96	2.14	2.74^B–D^	20.8^a–e^	16.9^f–j^	9.90^p–s^	15.9^BC^
*B*. *amyloliquefaciens* + 1BC	8.06^ab^	6.33^a–j^	3.81^l–u^	6.07^AB^	3.28	3.18	2.51	2.99^A–C^	21.3^a–c^	17.0^f–j^	11.9^n–r^	16.7^A–C^
2BC	7.95^a–c^	5.87^c–l^	3.62^o–u^	5.81^AB^	3.35	3.00	2.52	2.96^A–D^	21.5^a–e^	17.9^c–j^	11.1^o–r^	16.8^A–C^
*L*. *adecarboxylata* + 2BC	8.13^a^	7.18^a–g^	4.00^k–t^	6.43^AB^	3.48	3.13	2.47	3.03^A–C^	22.7^a^	18.2^c–i^	12.0^n–r^	17.6^AB^
*A*. *fabrum* + 2BC	7.90^a–d^	6.92^a–h^	4.51^j–s^	6.44^AB^	3.52	3.19	2.82	3.18^AB^	22.8^a^	18.9^b–g^	12.8^l–q^	18.2^A^
*P*. *aeruginosa* + 2BC	7.73^a–e^	7.48^a–f^	4.17^k–t^	6.46^AB^	3.55	3.29	2.23	3.02^A–C^	22.4^ab^	17.6^d–j^	11.9^n–r^	17.3^A–C^
*B*. *amyloliquefaciens* + 2BC	7.58^a–f^	7.14^a–g^	5.79^e–n^	6.84^A^	3.58	3.38	2.94	3.30^A^	22.9^a^	17.9^c–j^	13.2^k–p^	18.0^A^
ME (D)	6.87^A^	5.25^B^	3.35^C^		3.22^A^	2.93^B^	2.31^C^		19.7^A^	15.5^B^	10.6^C^	

Means sharing different letters are significantly different (*p* ≤ 0.05). Non-significant interactive effect (T × D) did not have any letter.

ME indicates main effect; IE indicates interactive effect; NM = Normal Moisture; MD = Mild Drought; SD = Severe Drought.

### N, P and K in grains

Both main and interactive effects of T and D were significantly different for N, P and K concentration in grain. At SD, the *L*. *adecarboxylata*, *A*. *fabrum*, *P*. *aeruginosa* and *B*. *amyloliquefaciens* with and without 1BC and 2BC differed significantly as compared to the control for grain nitrogen (Table 5). The *B*. *amyloliquefaciens*, 1BC, *L*. *adecarboxylata* + 1BC, *A*. *fabrum* + 1BC, *P*. *aeruginosa* + 1BC and *B*. *amyloliquefaciens* + 1BC, 2BC, *L*. *adecarboxylata* + 2BC, *A*. *fabrum* + 2BC, *P*. *aeruginosa* + 2BC and *B*. *amyloliquefaciens* + 2BC remained significantly better as compared to the control for grain nitrogen at MD. Application of *A*. *fabrum* + 2BC proved significantly better as compared to the control for grain nitrogen at NM. For grain phosphorus, all the treatments (except *P*. *aeruginosa*) were significantly different as compared to the control at SD. However, at MD and NM all the treatments were statistically similar to the control for grain P. In case of grain K, all the treatments were significantly different as compared to the control at SD. The *A*. *fabrum* and *P*. *aeruginosa* at 2BC remained significantly better than at 1BC for grain K at SD. At MD, inoculation of PGPR with 1BC and 2BC proved significantly better than the control for grain K. The performance of *A*. *fabrum* was significantly better at 2BC than at1BC for grain K at MD. Among all the treatments *A*. *fabrum* + 1BC, *B*. *amyloliquefaciens* + 1BC, 2BC, *L*. *adecarboxylata* + 2BC, *A*. *fabrum* + 2BC, *P*. *aeruginosa* + 2BC, *B*. *amyloliquefaciens* + 2BC remained significantly better at NM. The 2BC proved significantly better than 1BC for grain K at NM, MD and SD. Maximum increase in grain N (58%), P (18%) and K (23%) was noted through *B*. *amyloliquefaciens* + 2BC as compared to the control at SD.

**Table 5:** Effect of ACC deaminase containing PGPR in combination with various rates of timber waste biochar (0BC, 1BC and 2BC) on grains N, P and K concentration under various levels of drought (D).

Treatments	Grain Nitrogen (%)				Grain Phosphorus (%)				Grain Potassium (%)			
	Various levels of drought (D)											
	IE (T × D) (Means of 3 replicates)			ME (T)	IE (T × D) (Means of 3 replicates)			ME (T)	IE (T × D) (Means of 3 replicates)			ME (T)
	NM	MD	SD		NM	MD	SD		NM	MD	SD	
Control_(No PGPR+No BC)_	1.78^b–k^	1.56^no^	1.11^p^	1.48^G^	0.305^a–i^	0.283^i–n^	0.247^o^	0.278^D^	0.465^k–m^	0.453^mn^	0.422^n^	0.447^H^
*L*. *adecarboxylata*	1.77^c–m^	1.67^h–n^	1.49^o^	1.64^F^	0.303^a–j^	0.287^e–n^	0.273^mn^	0.288^B–D^	0.481^h–m^	0.474^h–m^	0.460^1–m^	0.472^G^
*A*. *fabrum*	1.79^b–k^	1.69^g–n^	1.56^no^	1.68^EF^	0.306^a–g^	0.299^b–l^	0.275^mn^	0.293^A–C^	0.491^f–l^	0.481^h–m^	0.460^l–m^	0.478^FG^
*P*. *aeruginosa*	1.80^b–j^	1.67^h–n^	1.56^no^	1.68^EF^	0.301^a–k^	0.280^j–n^	0.268^no^	0.283^CD^	0.482^h–m^	0.471^j–m^	0.455^m^	0.469^G^
*B*. *amyloliquefaciens*	1.79^b–j^	1.74^e–m^	1.60^m–o^	1.71^D–F^	0.309^a–g^	0.279^k–n^	0.274^mn^	0.287^B–D^	0.493^f–k^	0.484^g–m^	0.461^l–m^	0.479^E–G^
1BC	1.82^a–i^	1.78^b–l^	1.61^l–o^	1.74^C–E^	0.307^a–g^	0.280^j–n^	0.280^j–n^	0.289^B–D^	0.505^d–h^	0.489^f–l^	0.470^j–m^	0.488^EF^
*L*. *adecarboxylata* + 1BC	1.87^a–f^	1.77^c–m^	1.62^k–o^	1.75^B–E^	0.310^a–e^	0.283^i–n^	0.277^l–n^	0.290^BC^	0.506^d–h^	0.497^f–j^	0.481^h–m^	0.495^DE^
*A*. *fabrum* + 1BC	1.86^a–g^	1.78^c–l^	1.61^l–o^	1.75^B–E^	0.307^a–h^	0.283^i–n^	0.280^k–n^	0.290^BC^	0.535^a–d^	0.503^d–i^	0.473^i–m^	0.504^CD^
*P*. *aeruginosa* + 1BC	1.86^a–f^	1.78^b–l^	1.64^j–o^	1.76^B–E^	0.309^a–f^	0.283^i–n^	0.274^mn^	0.289^B––D^	0.520^b–f^	0.497^f–j^	0.465^k–m^	0.494^DE^
*B*. *amyloliquefaciens* + 1BC	1.91^a–d^	1.82^a–i^	1.66 ^i–o^	1.80^A–C^	0.312^a–d^	0.287^e–n^	0.279^k–n^	0.293^BC^	0.542^a–c^	0.519^b–f^	0.498^f–j^	0.520^B^
2BC	1.92^a–d^	1.81^a–i^	1.66^i–o^	1.80^A–D^	0.321^ab^	0.286^g–n^	0.276^mn^	0.294^A–C^	0.543^a–c^	0.532^a–e^	0.502^e–i^	0.526^AB^
*L*. *adecarboxylata* + 2BC	1.94^a–c^	1.84^a–h^	1.67^h–n^	1.82^A–C^	0.315^a–c^	0.290^d–n^	0.287^f–n^	0.297^AB^	0.550^ab^	0.519^b–f^	0.484^g–m^	0.517^BC^
*A*. *fabrum* + 2BC	1.97^a^	1.88^a–f^	1.71^f–n^	1.85^A^	0.318^ab^	0.293^c–m^	0.279^k–n^	0.296^AB^	0.560^a^	0.541^a–c^	0.515^c–g^	0.539^A^
*P*. *aeruginosa* + 2BC	1.90^a–e^	1.82^a–i^	1.67^h–n^	1.80^A–C^	0.309^a–g^	0.290^d–n^	0.285^h–n^	0.295^AB^	0.543^a–c^	0.531^a–e^	0.515^c–g^	0.530^AB^
*B*. *amyloliquefaciens* + 2BC	1.95^ab^	1.78^c–l^	1.75^d–m^	1.82^AB^	0.322^a^	0.299^a–l^	0.291^d–n^	0.304^A^	0.559^a^	0.541^a–c^	0.518^c–f^	0.539^A^
ME (D)	1.86^A^	1.76^B^	1.59^C^		0.310^A^	0.287^B^	0.276^C^		0.518^A^	0.502^B^	0.479^C^	

Means sharing different letters are significantly different (*p* ≤ 0.05). Non-significant interactive effect (T × D) did not have any letter.

ME indicates main effect; IE indicates interactive effect; NM = Normal Moisture; MD = Mild Drought; SD = Severe Drought.

### Discussion

In the current study, reduction in shoot length of wheat at MD and SD in the control might be due to competition for water and nutrients between root and shoot. Gargallo-Garriga *et al*.^40^ stated that drought stress deactivated shoot metabolic activity to conserve water and food which would have facilitated roots elongation. The reduction in water and nutrients movement in shoot, enhanced the up-regulation of ethylene precursor 1-aminocyclopropane-1-carboxylic acid (ACC)^41^ while significant amount of ACC generated ethylene restricted the root elongation^12^. Glick *et al*.^11^ suggested the mechanism for reduction in stress ethylene through the activity of ACC-deaminase^11^. According to Glick *et al*.^11^, the synthesis of indole acetic acid (IAA) by PGPR stimulates the elongation of plants cells and activates ACC synthase which converts S-adenosyl methionine to ACC. A significant amount of ACC is exuded by plants roots and seeds in rhizosphere which is hydrolyzed by ACC deaminase into NH_3_ and α-ketobutyrate, resulting in better roots elongation. Secretion of roots exudates (phytosiderophores, sugars, organic acids, amino acids, vitamins, nucleosides and mucilage) also attracts PGPR that colonize roots and facilitates better uptake of water and nutrients^42,43^. Besides imperative role of PGPR, high surface area, ion exchange capacity, nutrients and water holding capacity of BC make it an effective amendment for better intake of nutrients and water in plants^44–46^. A significant improvement in photosynthetic rate, transpiration rate and stomatal conductance under MD and SD, signified the effectiveness of co-application of *L*. *adecarboxylata*, *A*. *fabrum*, *P*. *aeruginosa*, *B*. *amyloliquefaciens* with 2BC, comparative to their sole application and the control. The increase in gas exchange attributes might be due to the better uptake of water and nutrients, improvement in soil water holding capacity (WHC), PGPR colonization and reduction in ethylene through the co-application of 2BC^47,48^ and ACC deaminase containing PGPR^49,50^. According to Zheng *et al*.^51^ and Borch *et al*.^52^ the deficiency of nitrogen and phosphorus significantly decrease the growth of crops. Siddique *et al*.^53^ suggested the less stomatal conductance as main cause of reduction in rate of transpiration under drought. Tholen *et al*.^54^ argued that drought stress decreased the intake of CO_2_ due to less stomatal conductance which restricted the carboxylation. Hence reduced the rate of photosynthesis^55^. However, A significant improvement in chlorophyll a, chlorophyll b, total chlorophyll, shoot and grain nutrients concentration validated the efficacious functioning of co-application of ACC deaminase producing PGPR and 2BC that also significantly increased the yield attributes (100-grains weight, straw yield and grains yield pot^−1^) of wheat plants at MD and SD. Richardson *et al*.^56^ and Zahir *et al*.^57^, suggested better roots elongation and secretion of organic acids by PGPR for P and K solubilization as key factors, responsible for better nutrients uptake, improvement in dry weight and yield of crops. Moreover, the biochar ability to sorb nutrients also reduced the losses of N and improved its uptake in plants^58,59^. Chan *et al*.^60^ suggested that the high surface area of biochar is a basic reason for improved cation exchange sites in soil which resulted in better bioavailability of nutrients. Results of the current study also showed that electrolyte leakage in the wheat plants leaves was decreased where *L*. *adecarboxylata*, *A*. *fabrum*, *P*. *aeruginosa*, *B*. *amyloliquefaciens* with 2BC were applied comparative to the control under MD and SD. The reduction in electrolyte leakage might be due to the activity of ACC deaminase, better availability of water and nutrients by co-application of PGPR and 2BC. Senaratna and McKersie^61^ observed a significant increase in electrolyte leakage because of cell membrane damage by drought stress which made it more permeable. According to Matile *et al*.^62^, cell usually lost its membrane integrity as a result of lipid degradation by ethylene. When the lipids in cell membrane become degraded, the ethylene comes in contact with the chloroplast and activates the chlorophyllase (chlase) gene which causes severe damage to the chlorophyll^62^. However, the addition of 2BC and PGPR in combination significantly decreased the electrolyte leakage which might be due to activity of ACC deaminase, better availability of water and nutrients uptake.

### Conclusion

It is concluded that the co-application of drought tolerant ACC deaminase producing PGPR and 2BC is comparatively better approach than their sole application to mitigate drought stress in wheat. Though *Leclercia adecarboxylata* and *Pseudomonas aeruginosa* were also effective enough but *Agrobacterium fabrum* and *Bacillus amyloliquefaciens* with 2BC gave maximum increase in the gas exchange attributes, nutrients concentration in shoot and grain, photosynthetic pigments and yield of wheat. More investigations are needed at field level to introduce *A*. *fabrum* and *B*. *amyloliquefaciens* with 2BC to improve growth and yield of wheat under drought stress.

## Materials and Methods

Out of 23 initially screened rhizobacteria (Table 7) isolated from wheat rhizospheric soil, collected from Old Shujabad Road (30.11°N and 71.43°E) and Akramabad (30.16°N and 71.29°E), four most efficient drought-tolerant ACC-deaminase producing PGPR were screened out after a laboratory hydroponic trial in the Department of Soil Science, Bahauddin Zakariya University Multan, Pakistan. For screening of most effective drought tolerant PGPR strains, polyethylene glycol 6000 (PEG-6000) was used (0, 10 and 20%) to maintain osmotic potential (0.05, −0.23 and −0.78 MPa) to introduce drought stress^63,64^.

Molecular identification of the most efficient drought tolerant ACC deaminase producing PGPR was done by 16S rRNA gene sequencing using PCR primers 1492R 5′ (TAC GGY TAC CTT GTT ACG ACT T) 3′ and 27F 5′ (AGA GTT TGA TCM TGG CTC AG) 3′. The gene sequencing primers were 907R 5′ (CCG TCA ATT CMT TTR AGT TT) 3′ and 785F 5′ (GGA TTA GAT ACC CTG GTA) 3′. Finally, 16S rRNA gene sequences were aligned and relationships were deduced using BLAST analysis^65^. Most efficient drought-tolerant ACC-deaminase producing PGPR were identified as AbW_1_ = *Leclercia adecarboxylata* (NR_104933.1), CbW_2_ = *Agrobacterium fabrum* (NR_074266.1), CbW_3_ = *Bacillus amyloliquefaciens* (FN_597644.1) and AbW_5_ = *Pseudomonas aeruginosa* (CP012001.1). These PGPR strains were able to grow at the osmotic potential −0.78 MPa generated through 20% polyethylene glycol 6000 (PEG). The DF minimal salt medium (4.0 g KH_2_PO_4_, 6.0 g Na_2_HPO_4_, 0.2 g, MgSO_4_.7H_2_O, 2.0 g glucose, 2.0 g gluconic acid and 2.0 g citric acid with trace elements: 1 mg FeSO_4_.7H_2_O, 10 mg H_3_BO_3_, 11.19 mg MnSO_4_.H_2_O, 124.6 mg ZnSO_4_.7H_2_O, 78.22 mg CuSO_4_.5H_2_O, 10 mg MoO_3_, pH = 7.2 and 0.5 M ACC as a sole nitrogen source) was used to grow the strains^66^. For determination of indole acetic acid (IAA) with and without L-tryptophan, Glickmann and Dessaux^67^ method was adopted.

To confirm the presence of AcdS gene that plays key role in synthesis of ACC deaminase NCBI gene bank was consulted. From NCBI gene bank it was confirmed that *B*. *amyloliquefaciens* (https://www.ncbi.nlm.nih.gov/nuccore/KX709841.1/), *A*. *fabrum* (https://www.ncbi.nlm.nih.gov/protein/PZP48640.1/) and *P*. *aeruginosa* (https://www.ncbi.nlm.nih.gov/nuccore/CP014948.1/) have AcdS gene while work is yet continued on *L*. *adecarboxylata*. For assessing the ACC deaminase produced by PGPR methodology of El-Tarabily^68^ and Honma and Shimomura^20^ was used. Pikovskaya’s medium was used to examine the phosphorus solubilizing activity of PGPR as described by Vazquez *et al*.^69^. Potassium solubilizing activity of PGPR was assessed according to the methodology of Candra Setiawati and Mutmainnah^70^. Characterization of the PGPR isolates is provided in Table 7.

For the production of biochar, timber waste was collected from local timber market. The timber waste was initially sun-dried and then pyrolyzed at 389 °C for 80 min in an especially designed pyrolyzer as described by Qayyum *et al*.^27^. All the pyrolyzed material (biochar) was then crushed in a grinder and passed through 2 mm sieve. Finally, the fine powder of timber waste biochar (BC) was stored in air tight plastic jars^27^.

The pH and EC*e* of BC were determined by mixing the BC and water with the ration, 1:20 (w/v) as described by Qayyum *et al*.^27^. Di-acid (HNO_3_: HClO_4_) digestion^71^ of biochar was done for the analysis of total phosphorus following yellow color method on spectrophotometer^72^, and those of potassium and sodium on flame photometer^73^. For the determination of nitrogen, H_2_SO_4_ digestion^72^ was done followed by distillation on Kjeldahl’s distillation apparatus^74^. The volatile matter and ash content of biochar were analyzed according to Qayyum *et al*.^75^ by heating the biochar in muffle furnace at 450 °C and 550 °C respectively. The fixed carbon in biochar was assessed (Table 6) using the equation as follows^76^;

1 $${\rm{Fixed}}\,{\rm{Carbon}}\,( \% )=100-( \% \,{\rm{Volatile}}\,{\rm{Matter}}+ \% \,{\rm{Ash}}\,{\rm{Content}})$$

The plastic bag (30 cm deep × 20 cm in diameter) was used as a pot, having capacity to carry 8 kg soil. The soil was collected from the plough layer of bank of the Chenab River, Multan, Punjab, Pakistan. The soil of selected area was previously characterized as dark yellowish brown, moderately calcareous, weakly structured and well drained with Cambic subsurface horizon and an Ochric epipedon^77^. The soil texture was determined by hydrometer method^78^ which was sandy loam (USDA triangle) with mixed hyperthermic Haplocambids. The organic matter in soil was determined by Walkley^79^. The organic nitrogen in soil was determined using the equation:

2 $${\rm{Organic}}\,{\rm{N}}\,( \% )={\rm{Soil}}\,{\rm{Organic}}\,{\rm{Matter}}/20$$

For extractable soil P determination, Olsen and Sommers^80^ method was used. Similarly, the extractable K in soil was determined according to the method described by Nadeem *et al*.^73^ (Table 6).

**Table 6:** Characteristics of soil, timber waste biochar (BC).

Experiment Soil			Biochar	Unit	Value
Attriburs	Unit	Value			
Sand	%	55	pH	—	7.03
Silt	%	30	EC_*e*_	dS m^−1^	0.89
Clay	%	15	Volatile Matter	%	30.26
Texture	Sandy Loam		Ash Content	%	10.19
pH_*s*_	—	8.43	Fixed Carbon	%	59.55
EC_*e*_	dS m^−1^	1.95	Total N	%	0.29
Organic Matter	%	0.45	Total P	%	0.53
Organic N	%	0.023	Total K	%	1.36
Extractable P	mg kg^−1^	8.16	Total Na	%	0.28
Extractable K	mg kg^−1^	204

**Table 7:** Characteristics of PGPR.

Isolates	PGPR traits				
	IAA without L-Tryptophan (µg/mL)	IAA with L-Tryptophan (µg/mL)	ACC deaminase (µmol α-ketobutyrate nmol mg^−1^ protein h^−1^)	Phosphorus solubilization (µg/mL)	Potassium solubilization (µg/mL)
BbW_6_	—	—	104.2 ± 10.9	—	12.4 ± 1.22
BbW_12_	—	—	94.9 ± 6.91	—	—
AbW_4_	0.86 ± 0.07	8.11 ± 1.26	131.3 ± 10.1	—	10.6 ± 1.91
CbW_4_	0.66 ± 0.12	9.52 ± 0.60	84.2 ± 7.19	6.22 ± 0.34	13.7 ± 1.63
AbW_1_	3.42 ± 0.27	67.8 ± 2.20	304.9 ± 24.1	26.6 ± 1.04	20.1 ± 1.02
BbW_9_	0.62 ± 0.06	7.33 ± 0.40	134.6 ± 20.6	10.2 ± 0.22	14.5 ± 1.58
AbW_9_	—	—	94.7 ± 15.3	—	9.84 ± 1.33
AbW_8_	0.16 ± 0.04	2.14 ± 0.17	181.2 ± 259	9.41 ± 0.29	11.7 ± 1.26
CbW_3_	1.12 ± 0.60	17.3 ± 2.34	313.2 ± 34.3	20.9 ± 2.48	23.4 ± 1.92
AbW_16_	—	—	144.3 ± 23.2	11.2 ± 0.12	15.6 ± 1.20
CbW_5_	—	—	153.5 ± 21.7	10.6 ± 0.27	13.3 ± 1.18
CbW_2_	2.43 ± 0.34	58.8 ± 3.27	349.6 ± 21.4	16.2 ± 1.48	26.7 ± 1.49
BbW_14_	—	—	149.6 ± 11.1	9.84 ± 0.10	14.7 ± 1.38
AbW_3_	—	—	209.2 ± 29.4	—	11.9 ± 1.61
BbW_8_	0.12 ± 0.04	3.44 ± 0.37	179.3 ± 26.8	8.21 ± 0.38	—
AbW_20_	—	—	172.0 ± 20.1	11.4 ± 0.22	13.6 ± 1.73
CbW_6_	0.36 ± 0.02	1.52 ± 0.35	159.6 ± 31.3	7.43 ± 0.19	15.2 ± 1.56
AbW_5_	3.16 ± 0.21	24.8 ± 1.49	245.4 ± 19.5	22.8 ± 1.36	17.9 ± 1.02
BbW_4_	0.56 ± 0.11	6.14 ± 1.06	349.6 ± 21.4	—	11.6 ± 1.44
BbW_10_	—	—	119.7 ± 24.9	13.4 ± 0.24	—
AbW_11_	—	—	194.7 ± 10.6	12.8 ± 0.29	—
AbW_2_	0.76 ± 0.05	14.7 ± 1.09	129.6 ± 7.46	13.0 ± 0.35	10.9 ± 1.41
CbW_7_	0.46 ± 0.09	10.4 ± 1.16	89.4 ± 10.1	11.9 ± 0.12	10.3 ± 1.28

In each plastic pot, 8 kg soil was filled. To fulfil macro nutrients requirement nitrogen (N), phosphorus (P) and potassium (K) fertilizers were added at the rate of 120: 90 and 60 kg ha^−1^ respectively, as recommended dose keeping in mind the nutrients concentration of biochar where it was applied^81^. The urea was added in three split doses. As far as diammonium phosphate (DAP) and muriate of potash (MOP) fertilizers are concerned, the recommended rates of fertilizers were applied in a single dose at the time of sowing. Timber waster biochar was added at three different rates including: control i.e., no biochar (0BC), 0.75% of soil (60 g biochar per 8 Kg soil) biochar (1BC) and 1.50% of soil (120 g biochar per 8 Kg soil) biochar (2BC).

The seeds of wheat (Galaxy-2013) were obtained from the certified seed dealer of the Government of Punjab, Pakistan. Healthy seeds were separated from broken and weak seeds. The seeds were surface-sterilized with sodium hypochlorite (5%) followed by 3 washes with ethanol (95%). Finally, all the seeds were washed three times with sterilized deionized water^82^. For PGPR inoculation, 10 ml of inoculum (0.5 optical density at 535 nm wavelength)^83^ was added along 10% sugar (glucose) in 100 g sterilized seeds. After proper mixing of seeds, inoculum and sugar solution, top dressing of seeds was done with a mixture of peat and clay (3:1 ratio) as described by Ahmad *et al*.^84^. Before inoculation of seeds, the peat and clay mixture was sterilized at 121 °C for 20 min in an autoclave^83^. All the control treatment seeds were also top dressed with peat and clay mixture along with 10% sugar solution without inoculum^85^.

In each pot, 10 seeds of wheat were initially sown. In control, the soil normal moisture (NM) was maintained at the level of 70% of field capacity (FC_70_) throughout the experiment on weight basis. However, to introduce mild drought (MD) and severe drought (SD) stress as per treatment plan, the soil moisture was maintained at the level of 50% and 30% of field capacity (FC_50_ and FC_30_), respectively, throughout the trial as suggested by Boutraa *et al*.^86^. After germination of seeds, five healthy seedlings were kept in each pot by thinning.

The pot experiment was conducted in the research area of the Department of Soil Science, Bahauddin Zakariya University Multan, Pakistan under drought stress on wheat. There were 15 treatments with 3 replications, following factorial completely randomized design (CRD). The treatments included: Control (No PGPR + No BC), *L*. *adecarboxylata*, *A*. *fabrum*, *P*. *aeruginosa*, *B*. *amyloliquefaciens*, 1BC, *L*. *adecarboxylata* + 1BC, *A*. *fabrum* + 1BC, *P*. *aeruginosa* + 1BC, *B*. *amyloliquefaciens* + 1BC, 2BC, *L*. *adecarboxylata* + 2BC, *A*. *fabrum* + 2BC, *P*. *aeruginosa* + 2BC and *B*. *amyloliquefaciens* + 2BC.

Leaf gas exchange parameters (net photosynthetic rate, net transpiration rate and stomatal conductance) were determined with the help of Infra-Red Gas Analyzer (CI-340 Photosynthesis system, CID, Inc. USA) by joining 4 leaves of wheat. On a sunny day, the readings were taken between 10:30 and 11:30 AM at saturating intensity of light^87^.

After 50 days of sowing, the seedlings were harvested from each pot for the measurement of shoot length and determination of electrolyte leakage, proline contents, photosynthetic pigments level and nutrients concentration in the shoot.

The electrolyte leakage (EL) was determined following the procedure given by Lutts *et al*.^88^. The leaves were washed with deionized water and then cut using a steel cylinder having diameter 1 cm. One gram of uniform sized leaf pieces were immersed in a test tube containing deionized water (20 ml) and incubated at 25 °C for 24 h. The electrical conductivity (EC1) was determined using pre-calibrated EC meter. The second EC (EC2) was noted heating the test tubes in a water bath at 120 °C for 20 min. The final value of EL was calculated using the equation as follows;

3 $${\rm{Electrolyte}}\,{\rm{Leakage}}\,({\rm{EL}})={\rm{EC}}1/{\rm{EC}}2\times 100$$

For proline assessment in wheat leaves, methodology stated by Bates *et al*.^89^ was followed. The proline was extracted from fresh (0.1 g) leaves in 2 ml of 40% methanol. After extraction, the 1 ml mixture of glacial acetic acid and 6 M orthophosphoric acid (3:2 v/v) was mixed in 1 ml extract along with 25 mg ninhydrin. Then the solution was incubated at 100 °C for 60 min. After cooling down, 5 ml Toluene was added. For the estimation of proline contents, absorbance was noted on spectrophotometer at 520 nm wavelength.

The chlorophyll a, chlorophyll b and total chlorophyll contents were determined in the fresh leaves of wheat according to the protocol given by Arnon^90^. The extract was taken from the leaves using acetone (80%) solution. For the estimation of chlorophyll a and chlorophyll b, the absorbance was taken at 663 and 645 nm wavelength, respectively on spectrophotometer. Final calculations were made using the following relations;

4 $${\rm{Chlorophyll}}\,{\rm{a}}\,({\rm{mg}}/{\rm{g}})=12.7\,({\rm{OD}}\,663)\,\mbox{--}\,2.69\,({\rm{OD}}\,645)\,{\rm{V}}/1000\,({\rm{W}})$$

5 $${\rm{Chlorophyll}}\,{\rm{b}}\,({\rm{mg}}/{\rm{g}})=22.9\,({\rm{OD}}\,645)\,\mbox{--}\,4.68\,({\rm{OD}}\,663){\rm{V}}/1000\,({\rm{W}})$$

6 $${\rm{Total}}\,{\rm{Chlorophyll}}\,({\rm{mg}}/{\rm{g}})={\rm{Chlorophyll}}\,{\rm{a}}+{\rm{Chlorophyll}}\,{\rm{b}}$$

where, OD = Optical density (wavelength). V = Final volume made. W = Fresh leaf made (g).

The samples were digested with sulfuric acid^72^ followed by distillation on Kjeldahl’s distillation apparatus^74^. The yellow colour method was used for the determination of phosphorus concentration noting absorbance at 420 nm on spectrophotometer^72^. As far as the K concentration in wheat shoot and grain is concerned, the samples were digested and then run on flame photometer as described by Nadeem *et al*.^73^.

The wheat plants were harvested after 125 days of sowing for the determination of grains yield pot^−1^, straw yield pot^−1^ and 100-grain weight. Weight of 100 grains, straw and grains yield pot^−1^ were assessed on top weight balance. For straw yield, plants were harvested at 4 inches above the ground surface. Sun dried 100 grains of wheat were counted randomly and manually and then weighed on top weight balance. Total wheat grains collected from a single pot were weighed and considered as grain yield pot^−1^.

Statistical analyses of the data were carried out using standard statistical procedures^91^. All the treatments were compared using Tukey’s test at *p* ≤ 0.05.

## Data Availability

No datasets were generated or analyzed during the current study. All the analyzed data can be accessed after publication by requesting the corresponding author.
